# Supplementary figures and images for: Investigation of cell signalings and therapeutic targets in PTPRK-RSPO3 fusion-positive colorectal cancer
Source: PLoS One. 2022 Sep 21;17(9):e0274555. doi: 10.1371/journal.pone.0274555 (PMC9491571; doi:10.1371/journal.pone.0274555)

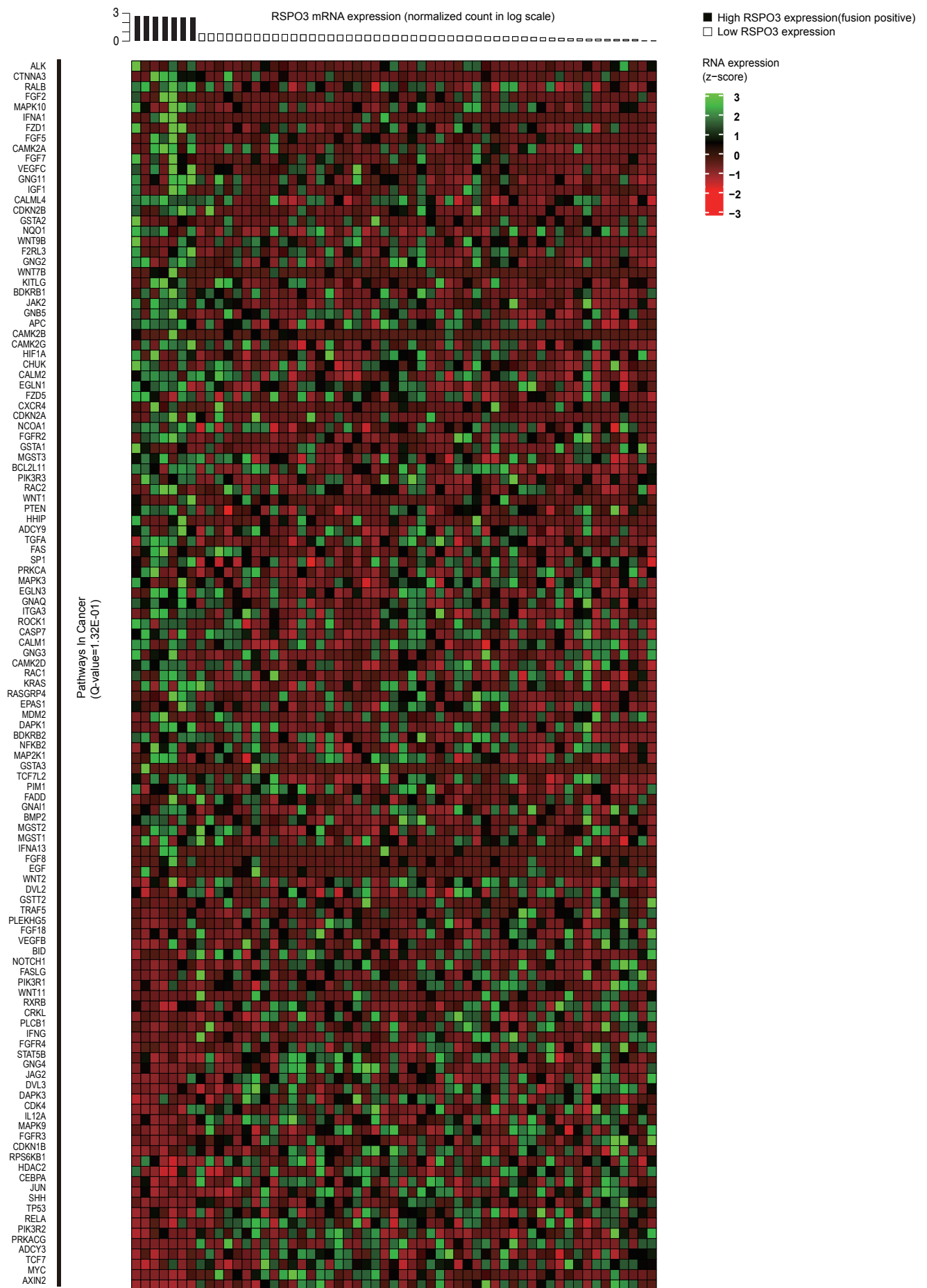

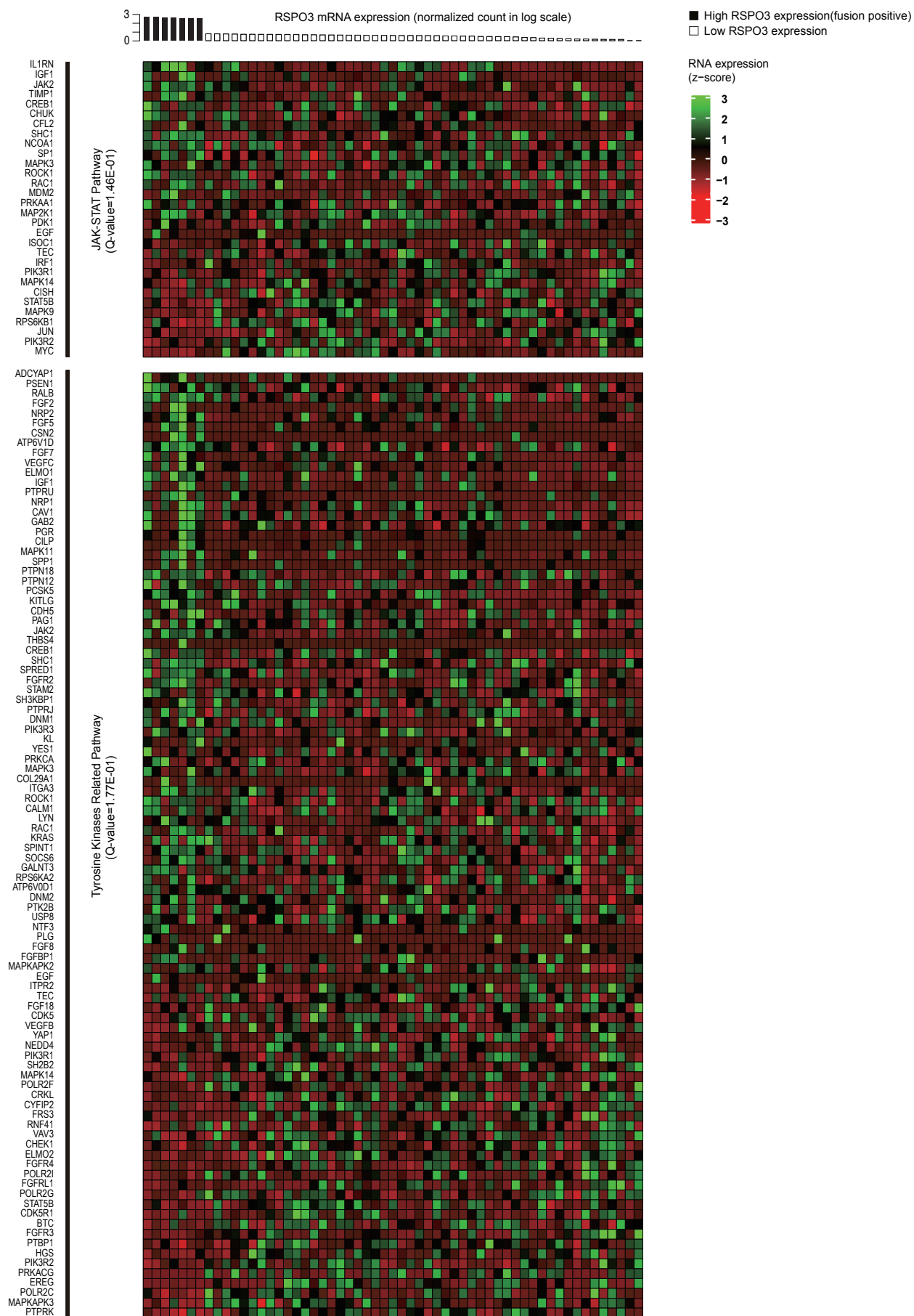

Supplement: S1 Fig — (PDF) [file pone.0274555.s001.pdf]

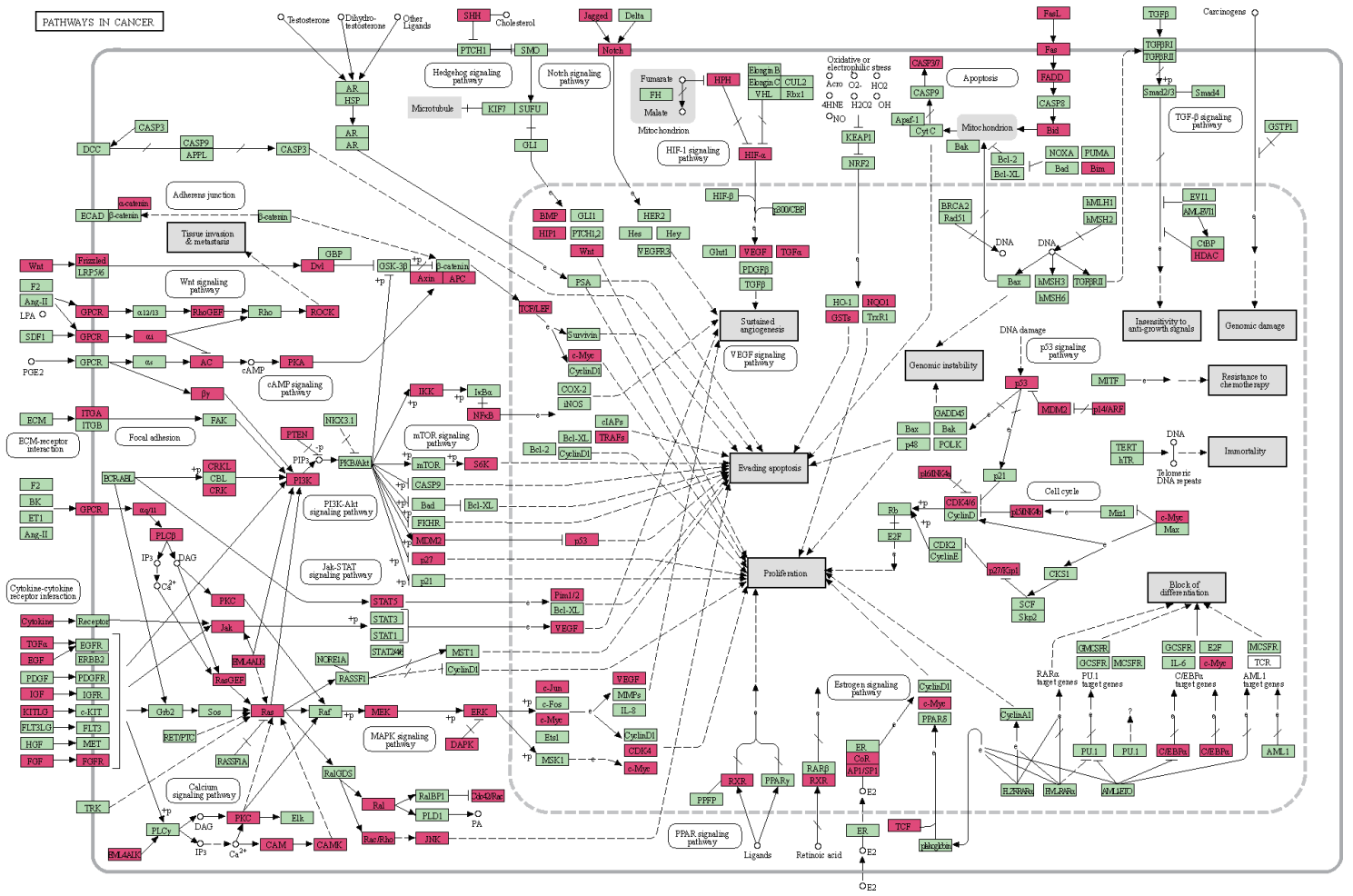

ERBB SIGNALING PATHWAY

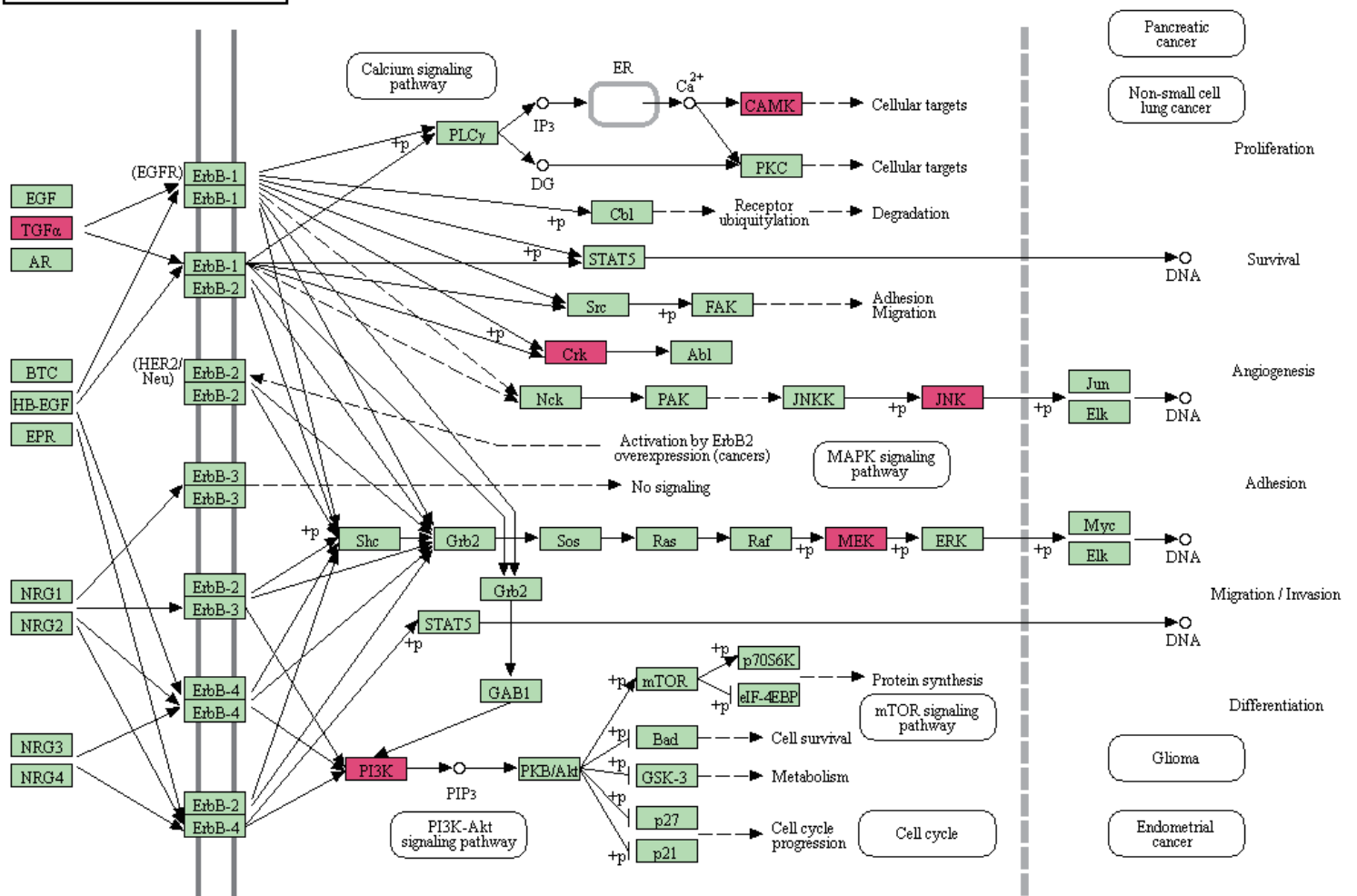

Supplement: S2 Fig — (PDF) [file pone.0274555.s002.pdf]

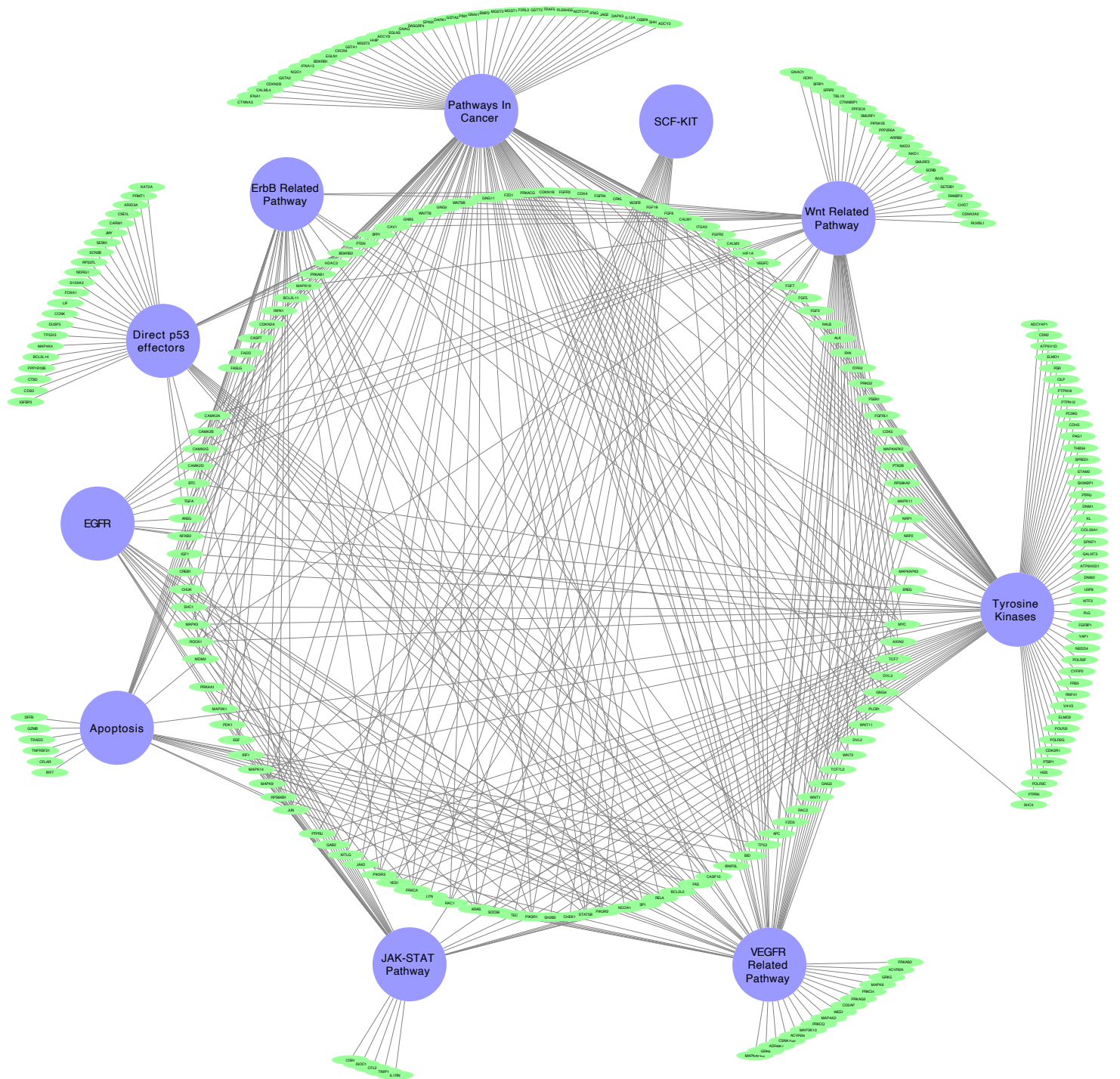

Supplement: S3 Fig — (PDF) [file pone.0274555.s003.pdf]

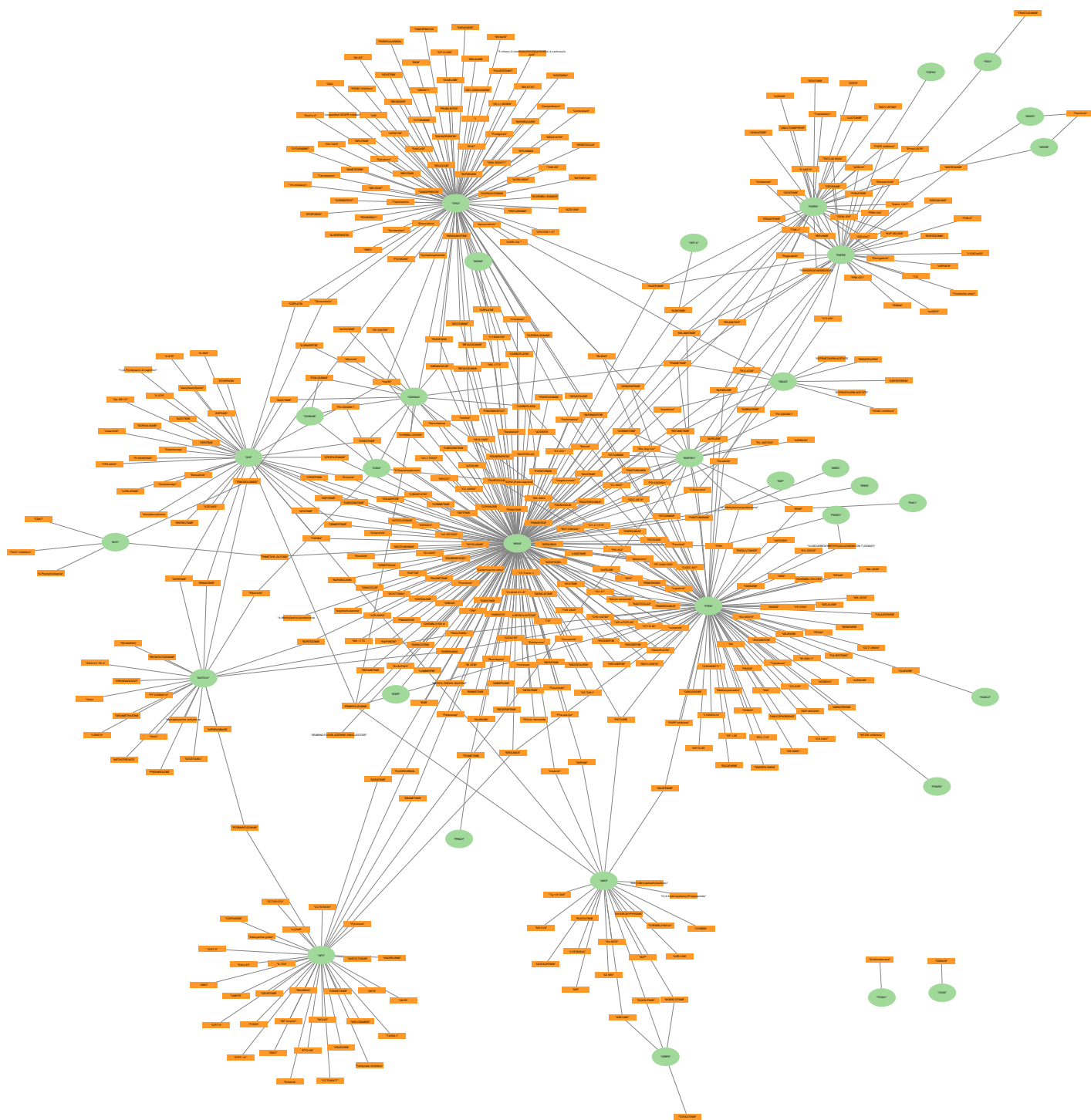

Supplement: S4 Fig — (PDF) [file pone.0274555.s004.pdf]
