# Supplementary material for: Investigation of cell signalings and therapeutic targets in PTPRK-RSPO3 fusion-positive colorectal cancer
Source: PLoS One. 2022 Sep 21;17(9):e0274555. doi: 10.1371/journal.pone.0274555 (PMC9491571; doi:10.1371/journal.pone.0274555)
Supplement: Attachment — Submitted filename: Response to Reviewers.docx [file pone.0274555.s007.docx]

Response to Reviewer Comments

We deeply appreciate the reviewer Meysam Sarshar’s attention to our paper. Below, we address each of the reviewer's comments point by point. We revised our manuscript using MS Word and highlighted the changes with yellow color.

**Point 1**: Line 82: the following sentences are not clear. 372 tumor samples with the barcode 01A were chosen, with other types of tumor samples, 11A (Normal) or 06A (Metastasized), being excluded. What do these numbers and barcodes referred to?

**Response 1**: Thank you for your comment. For better understanding of the TCGA data for readers, the following sentence has been modified as follows (Line: 78-79):

“Of the 433 GDAC data downloaded, 53 normal and 1 metastasis data were omitted, and the remaining 379 data were analyzed.”

The barcode contains information concerning the institution, patient, sample, and method of analysis. For instance, the two digits from 01A, 06A, 11A barcodes reveal information on the type of patient sample whether it be normal or tumor, and the alphabet indicates the vial containing the patient sample (https://gdc.cancer.gov/resources-tcga-users/tcga-code-tables/sample-type-codes). Although the majority of the TCGA data were tumor samples including 01-09, a portion of them, 10-19 were normal samples. Therefore, it was necessary to select and assess samples according to the purpose of analysis. We extracted primary solid tumor samples, which have barcodes 01, used for this study.

**Point 2**: How was the permutation test calculated? As stated by the authors, the median of absolute R values, mostly affected 2,505 genes were selected by correlation cut-off (R > 0.2). What is the criteria to set a cut-off of R > 0.2?

**Response 2**: Thank you for your detailed comment that improving our study. Of the 57 samples, we selected two genes and their RNA expression values were randomly ordered respectively, and Pearson correlation coefficient R value was obtained between two genes. This process was repeated 100,000 times. The resulting frequency of an R value higher than 0.2 by chance was 378 out of 100,000 times. Among the 2,505 genes that showed a correlation with RSPO3 of an R value of 0.2 or higher, approximately 0.378%, that is 20,531 gene multiplied by 0.00378 were estimated to yield 78 false positives. Hence, of the 2,505 genes, 2,427 genes may have a correlation with RSPO3.

**Point 3:** What does median in Table S1 imply? (Moreover, what does median in Table S1 imply?)

**Response 3**: Thank you for your comment. For instance, should KRAS mutation-positive samples occupy a majority of the 50 samples that were selected as control, there is a high likelihood for a large number of KRAS signature to be generated. In order to minimize the bias from undesired signature accumulation, 50 samples from the 186 samples expressing low RSPO3 expression were randomly selected as the control. Pearson R values were calculated between reference gene (RSPO3) and the other genes. Above process was repeated 100 times and the median R values were calculated in each gene.

**Point 4:** Line 128: As stated, 7 patients demonstrated presence of the fusion mutation, whereas the remaining 416 patients were negative for the fusion based on the clinicopathological characteristics. These cases are not fully presented in the Table 1. The characteristics of PTPRK-RSPO3 fusion-positive and fusion-negative cases in TCGA colorectal cancer is not clear in Table 1.

**Response 4**: Thank you for valuable comment. According to your comment, table 1 has been revised (Line: 138-140). The number of fusion and control samples were more clearly described in our study. The TCGA colorectal RNA samples used were extracted from the GDAC firehose. Of them, 379 were tumor solid samples, and of the 372 samples, excluding the 7 fusion-positive samples, 186 samples expressing less than 50% RSPO3 mRNA expression were selected as control (fusion-positive). The clinicopathological analysis was performed according to the criteria above and reflected in Table 1 and its pertinent text.

We modified the sentences on line 123-126 as follows:

“The clinicopathological characteristics in this study were described in Table 1. A total 379 tumor solid samples, and of the 372 samples, excluding the 7 fusion-positive samples, 186 samples expressing less than 50% RSPO3 mRNA expression were selected as control.”

**Point 5:** All the supplementary Tables and figures should be renamed as indicated in Sup section including tittle of each and applied methods for data extraction.

**Response 5**: Thank you for your comment. We revised the supplementary section on line 263-275 as follows:

S1 Fig: Gene expression heatmap of cancer-related pathways enriched with genes correlated to RSPO3 in RNA expression.

S2 Fig: The KEGG pathway maps for the human ERBB signaling pathway and pathways in cancer using the KEGG Mapper; genes correlated with RSPO3 expression are colored in pink.

S3 Fig: Putative target genes involved in multiple pathways of PTPRK-RSPO3 fusion-positive cancer.

S4 Fig: Inferred drug-target network in PTPRK-RSPO3 fusion-positive colorectal cancer based on VICC database.

S1 Table: 2,505 genes correlated with RSPO3 (R >0.2).

S2 Table: Putative target genes and actionable drugs involved in ten major cancer-related pathways.

**Point 6:** As Table 1 shows, none of the clinicopathological characteristics of selected cases is significant. Eighteen genes showed good correlation (R > 0.5) with RSPO3 in RNA expression. Is that correlation defines any significant RNA expression?

**Response 6:** Thank you for your comment. Since an overexpression of RSPO3 mRNA level is observed in PTPRK-RSPO3 fusion samples (PMID: 32631391 Figure 2B), we assumed that downstream genes affected by upregulated RSPO3 expression with fusion would have high R values in expression level by the Pearson correlation test. Due to the limited number (n=7) of RSPO3 fusion samples, it was not ideal to infer a statistically significant difference from clinical variable.

**Point 7:** Line 153: How come the ten different pathways were shown to be statistically significant? How was the significant means elaborated?

**Response 7:** Thank you for your comment. In summary, a P-value is calculated using the hypergeometric distribution. The P-value reflects the significance of the observed overlap between the input gene list and the module's members when compared to random expectations. A low P-value indicates that more of the module's members are present in the input list than would be expected by chance. The p-values are corrected for multiple testing using the false discovery rate method.

Detailed explanation: PMID: 18940869

**Point 8:** Line 163: the following sentence “ten genes from highest R-values were as follows” is not clear. What is the implication of highest R-values?

**Response 8:** Thank you for your comment. Since mRNA overexpression of RSPO3 occurs by PTPRK-RSPO3 fusion (PMID: 32631391 Figure 2B), it was determined that genes affected by expression downstream by upregulated RSPO3 would have high R values in the Pearson correlation test. The reason for selecting the 10 highest R-value genes is to find the most likely downstream pathway or target in the absence of a direct target therapy for RSPO3. Genes with the highest R values were extracted with the purpose of identifying therapeutic targets from the downstream pathway of RSPO3.

**Point 9:** Fig 4 represents Drug-target relation obtained based on Civic and OncoKB databases. What was the intention to select these two databases? The VICC meta-database has recently been reported the most extensive source of information, featuring 92% of variants with a drug association (https://dx.doi.org/10.21873%2Fcgp.20250). Is there any explanation?

**Response 9:** Thank you for your comment. The data provided by VICC meta-database is in the form of a JSON file. However, since the JSON files were broken and not unified in ordinary format, we had to manually parse every five databases (jax, brca, cgi, molecularmatch, pmkb) except CIViC and OncoKB. Then, we were only able to extract information about genes and drugs from jax, brca and molecularmatch databases. However, advanced information such as clinical significance, evidence direction and evidence type were limited to parse. For this reason, the analyzed results using VICC database were not included in the main figure but were included in the supplementary figures. We added explanation about VICC figures in our manuscript (Line: 270, 295).

**Point 10:** Line 184: 4 genes (KRAS, FGFR2, ALK, and JAK2) were identified and they were all included in the inferred results using CIViC database. How the authors link the gene presence with their RNA expression involved in several pathways as well as disease conditions?

**Response 10:** Thank you for your comment. Generally, there are three classes for activation signaling including hotspot mutation, amplification, and overexpression. For this study, RNA sequence-based overexpression was considered an activating signal. The drug database provides information on the relationship between the activating signaling (three classes mentioned above) and available drugs. For example, MET activating mutations are including amplification, over-expression, and activating point mutations and the three class of mutations are mostly sharing target-drug sensitivity (capmatinib, tepotinib). So, the three types of mutations were considered as showing similar target-drug sensitivity in silico level. Although the sensitivity of the drugs may differ according to the various types of signal activation, the purpose of this study is to enroll as many drugs with high potential as possible. It would be ideal for these hypotheses to be validated with further additional experimentations. However, the scope of this study does not encompass validation experiments and will take into consideration for future studies.

**Point 11:** Line 191: Of 19 druggable genes, five were involved in the multiple pathways. Have these genes as well as their related pathways experimentally reported?

**Response 11:** Thank you for your comment. While reviewing the content, five additional druggable genes involved multiple pathways were found and added to the manuscript (Line:217-230). The Consensus Path Database (CPDB, http://cpdb.molgen.mpg.de/CPDB) we utilized is a database combined by integrating 177 biological pathways based on experimentally reported studies: INOH (PMID: 2212066), KEGG (PMID: 27899662), NetPath (PMID: 20067622), PID (PMID: 18832364), Reactome (PMID: 26656494), and Wikipathways (PMID: 26481357). We attached a table of CPDB sources and 10 druggable genes involved in multiple pathways on below:

| **Gene** | **Source** | **Pathway** |
| --- | --- | --- |
| PIK3R1 | KEGG | Apoptosis |
| PIK3R1 | KEGG | VEGFR Related Pathway |
| PIK3R1 | KEGG | ErbB Related Pathway |
| PIK3R1 | KEGG | JAK-STAT Pathway |
| PIK3R1 | KEGG | EGFR |
| PIK3R1 | KEGG | Tyrosine Kinases |
| PIK3R1 | KEGG | Pathways In Cancer |
| PIK3R1 | KEGG | SCF-KIT |
| PIK3R1 | Reactome | Apoptosis |
| PIK3R1 | Reactome | VEGFR Related Pathway |
| PIK3R1 | Reactome | ErbB Related Pathway |
| PIK3R1 | Reactome | JAK-STAT Pathway |
| PIK3R1 | Reactome | EGFR |
| PIK3R1 | Reactome | Tyrosine Kinases |
| PIK3R1 | Reactome | Pathways In Cancer |
| PIK3R1 | Reactome | SCF-KIT |
| PIK3R1 | Wikipathways | Apoptosis |
| PIK3R1 | Wikipathways | VEGFR Related Pathway |
| PIK3R1 | Wikipathways | ErbB Related Pathway |
| PIK3R1 | Wikipathways | JAK-STAT Pathway |
| PIK3R1 | Wikipathways | EGFR |
| PIK3R1 | Wikipathways | Tyrosine Kinases |
| PIK3R1 | Wikipathways | Pathways In Cancer |
| PIK3R1 | Wikipathways | SCF-KIT |
| PIK3R1 | PharmGKB | Apoptosis |
| PIK3R1 | PharmGKB | VEGFR Related Pathway |
| PIK3R1 | PharmGKB | ErbB Related Pathway |
| PIK3R1 | PharmGKB | JAK-STAT Pathway |
| PIK3R1 | PharmGKB | EGFR |
| PIK3R1 | PharmGKB | Tyrosine Kinases |
| PIK3R1 | PharmGKB | Pathways In Cancer |
| PIK3R1 | PharmGKB | SCF-KIT |
| PIK3R1 | PID | Apoptosis |
| PIK3R1 | PID | VEGFR Related Pathway |
| PIK3R1 | PID | ErbB Related Pathway |
| PIK3R1 | PID | JAK-STAT Pathway |
| PIK3R1 | PID | EGFR |
| PIK3R1 | PID | Tyrosine Kinases |
| PIK3R1 | PID | Pathways In Cancer |
| PIK3R1 | PID | SCF-KIT |
| PIK3R1 | INOH | Apoptosis |
| PIK3R1 | INOH | VEGFR Related Pathway |
| PIK3R1 | INOH | ErbB Related Pathway |
| PIK3R1 | INOH | JAK-STAT Pathway |
| PIK3R1 | INOH | EGFR |
| PIK3R1 | INOH | Tyrosine Kinases |
| PIK3R1 | INOH | Pathways In Cancer |
| PIK3R1 | INOH | SCF-KIT |
| KRAS | Reactome | Pathways In Cancer |
| KRAS | Reactome | Tyrosine Kinases |
| KRAS | Reactome | ErbB Related Pathway |
| KRAS | Reactome | SCF-KIT |
| KRAS | Reactome | VEGFR Related Pathway |
| KRAS | Reactome | EGFR |
| KRAS | KEGG | Pathways In Cancer |
| KRAS | KEGG | Tyrosine Kinases |
| KRAS | KEGG | ErbB Related Pathway |
| KRAS | KEGG | SCF-KIT |
| KRAS | KEGG | VEGFR Related Pathway |
| KRAS | KEGG | EGFR |
| KRAS | Wikipathways | Pathways In Cancer |
| KRAS | Wikipathways | Tyrosine Kinases |
| KRAS | Wikipathways | ErbB Related Pathway |
| KRAS | Wikipathways | SCF-KIT |
| KRAS | Wikipathways | VEGFR Related Pathway |
| KRAS | Wikipathways | EGFR |
| KRAS | PharmGKB | Pathways In Cancer |
| KRAS | PharmGKB | Tyrosine Kinases |
| KRAS | PharmGKB | ErbB Related Pathway |
| KRAS | PharmGKB | SCF-KIT |
| KRAS | PharmGKB | VEGFR Related Pathway |
| KRAS | PharmGKB | EGFR |
| JAK2 | KEGG | SCF-KIT |
| JAK2 | KEGG | Tyrosine Kinases |
| JAK2 | KEGG | Pathways In Cancer |
| JAK2 | KEGG | VEGFR Related Pathway |
| JAK2 | KEGG | JAK-STAT Pathway |
| JAK2 | Reactome | SCF-KIT |
| JAK2 | Reactome | Tyrosine Kinases |
| JAK2 | Reactome | Pathways In Cancer |
| JAK2 | Reactome | VEGFR Related Pathway |
| JAK2 | Reactome | JAK-STAT Pathway |
| JAK2 | INOH | SCF-KIT |
| JAK2 | INOH | Tyrosine Kinases |
| JAK2 | INOH | Pathways In Cancer |
| JAK2 | INOH | VEGFR Related Pathway |
| JAK2 | INOH | JAK-STAT Pathway |
| JAK2 | Wikipathways | SCF-KIT |
| JAK2 | Wikipathways | Tyrosine Kinases |
| JAK2 | Wikipathways | Pathways In Cancer |
| JAK2 | Wikipathways | VEGFR Related Pathway |
| JAK2 | Wikipathways | JAK-STAT Pathway |
| TP53 | KEGG | Direct p53 effectors |
| TP53 | KEGG | ErbB Related Pathway |
| TP53 | KEGG | Wnt Related Pathway |
| TP53 | KEGG | Apoptosis |
| TP53 | KEGG | Pathways In Cancer |
| TP53 | Wikipathways | Direct p53 effectors |
| TP53 | Wikipathways | ErbB Related Pathway |
| TP53 | Wikipathways | Wnt Related Pathway |
| TP53 | Wikipathways | Apoptosis |
| TP53 | Wikipathways | Pathways In Cancer |
| TP53 | PID | Direct p53 effectors |
| TP53 | PID | ErbB Related Pathway |
| TP53 | PID | Wnt Related Pathway |
| TP53 | PID | Apoptosis |
| TP53 | PID | Pathways In Cancer |
| MAP2K1 | Wikipathways | JAK-STAT Pathway |
| MAP2K1 | Wikipathways | VEGFR Related Pathway |
| MAP2K1 | Wikipathways | Pathways In Cancer |
| MAP2K1 | Wikipathways | EGFR |
| MAP2K1 | Wikipathways | ErbB Related Pathway |
| MAP2K1 | KEGG | JAK-STAT Pathway |
| MAP2K1 | KEGG | VEGFR Related Pathway |
| MAP2K1 | KEGG | Pathways In Cancer |
| MAP2K1 | KEGG | EGFR |
| MAP2K1 | KEGG | ErbB Related Pathway |
| MAP2K1 | PharmGKB | JAK-STAT Pathway |
| MAP2K1 | PharmGKB | VEGFR Related Pathway |
| MAP2K1 | PharmGKB | Pathways In Cancer |
| MAP2K1 | PharmGKB | EGFR |
| MAP2K1 | PharmGKB | ErbB Related Pathway |
| MAP2K1 | INOH | JAK-STAT Pathway |
| MAP2K1 | INOH | VEGFR Related Pathway |
| MAP2K1 | INOH | Pathways In Cancer |
| MAP2K1 | INOH | EGFR |
| MAP2K1 | INOH | ErbB Related Pathway |
| FGFR2 | KEGG | Pathways In Cancer |
| FGFR2 | KEGG | VEGFR Related Pathway |
| FGFR2 | KEGG | Tyrosine Kinases |
| FGFR2 | Reactome | Pathways In Cancer |
| FGFR2 | Reactome | VEGFR Related Pathway |
| FGFR2 | Reactome | Tyrosine Kinases |
| FGFR2 | PharmGKB | Pathways In Cancer |
| FGFR2 | PharmGKB | VEGFR Related Pathway |
| FGFR2 | PharmGKB | Tyrosine Kinases |
| FGFR2 | INOH | Pathways In Cancer |
| FGFR2 | INOH | VEGFR Related Pathway |
| FGFR2 | INOH | Tyrosine Kinases |
| ALK | KEGG | VEGFR Related Pathway |
| ALK | KEGG | Pathways In Cancer |
| ALK | INOH | VEGFR Related Pathway |
| ALK | INOH | Pathways In Cancer |
| HIF1A | PID | VEGFR Related Pathway |
| HIF1A | PID | Pathways In Cancer |
| HIF1A | KEGG | VEGFR Related Pathway |
| HIF1A | KEGG | Pathways In Cancer |
| CDKN1B | KEGG | Pathways In Cancer |
| CDKN1B | KEGG | ErbB Related Pathway |
| CDKN1B | Wikipathways | Pathways In Cancer |
| CDKN1B | Wikipathways | ErbB Related Pathway |
| PTEN | KEGG | Direct p53 effectors |
| PTEN | KEGG | Pathways In Cancer |
| PTEN | PID | Direct p53 effectors |
| PTEN | PID | Pathways In Cancer |
| PTEN | Wikipathways | Direct p53 effectors |
| PTEN | Wikipathways | Pathways In Cancer |
